# A behavioral defect of temporal association memory in mice that partly lack dopamine reuptake transporter

**DOI:** 10.1038/srep17461

**Published:** 2015-12-10

**Authors:** Shining Deng, Lingli Zhang, Tailin Zhu, Yan-Mei Liu, Hailong Zhang, Yiping Shen, Wei-Guang Li, Fei Li

**Affiliations:** 1 Departments of Developmental and Behavioral Pediatrics, Medical Genetics, Shanghai Institute of Pediatric Translational Medicine, Shanghai Children’s Medical Center, Ministry of Education–Shanghai Key Laboratory of Children’s Environmental Health, Shanghai Jiao Tong University School of Medicine, Shanghai 200129; 2 Discipline of Neuroscience and Department of Anatomy, Histology and Embryology, Institute of Medical Sciences, Shanghai Jiao Tong University School of Medicine, Shanghai 200025, China

## Abstract

Temporal association memory, like working memory, is a type of episodic memory in which temporally discontinuous elements are associated. However, the mechanisms that govern this association remain incompletely understood. Here, we identify a crucial role of dopaminergic action in temporal association memory. We used hemizygote hyperdopaminergic mutant mice with reduced dopamine transporter (DAT) expression, referred to as DAT^+/−^ mice. We found that mice with this modest dopamine imbalance exhibited significantly impaired trace fear conditioning, which necessitates the association of temporally discontinuous elements, and intact delay auditory fear conditioning, which does not. Moreover, the DAT^+/−^ mice displayed substantial impairments in non-matching-to-place spatial working-memory tasks. Interestingly, these temporal association and working memory deficits could be mimicked by a low dose of the dopamine D2 receptor antagonist haloperidol. The shared phenotypes resulting from either the genetic reduction of DAT or the pharmacological inhibition of the D2 receptor collectively indicate that temporal association memory necessitates precise regulation of dopaminergic signaling. The particular defect in temporal association memory due to partial lack of DAT provides mechanistic insights on the understanding of cognitive impairments in multiple neurodevelopmental disorders.

Temporal association memory^1^ is a type of episodic memory that shares a critical feature with some forms of working memory^2^,^3^,^4^, namely, dependence on the ability to associate temporally discontinuous elements. The fundamental mechanisms of temporal association memory are likely shared with higher cognitive functions, and also may be affected in the pathophysiology^5^ of schizophrenia, attention-deficit hyperactivity disorder (ADHD), and Alzheimer’s disease^6^ due to their shared behavioral impairments on the particular aspect of cognition, as these disorders have shared memory impairments. As with working memory, mechanistic studies addressing the systems and circuit bases underlying the association process are emerging^1^,^7^,^8^, although the molecular and cellular details remain incompletely understood. Indeed, the characterization of the synaptic regulation that occurs during temporal association memory^1^,^9^ and working memory^10^ is necessary to further clarify the cognitive processes underlying association.

Dopamine is an important modulatory neurotransmitter that participates in complex brain functions, including the initiation and planning of motor activity, the identification of salient stimuli that predict rewards, and the spatiotemporal organization of goal-oriented behaviors^11^. The importance of the dopaminergic system^12^,^13^,^14^,^15^,^16^ has been greatly appreciated because of the motor, cognitive, emotional, and motivational deficits that constitute the hallmarks of multiple psychiatric and neurological disorders including ADHD, schizophrenia, Parkinson’s disease^17^, and depression^18^,^19^. Dopaminergic neurons originate from the ventral tegmental area^20^ and substantia nigra compacta^21^. From there they project to and activate dopamine D1- and D2-like receptors in nearly every brain region, including the prefrontal cortex, medial temporal lobe, and hippocampus, which are known to be actively involved in working memory^22^,^23^,^24^ and temporal association memory^1^,^7^,^9^. Notably, dopamine and its receptors are thought to be functionally critical for attention and working memory, which is mediated by brain regions such as the prefrontal cortex^22^,^23^,^24^. The separable roles of dopamine may be revealed *via* the genetically tractable organisms, however, no such study has addressed that. Moreover, the regulation of dopamine to which extent, in temporal association process, is yet to be established.

Acting as a primary cellular mechanism to terminate dopamine signaling, the dopamine transporter (DAT), which is located at the dopaminergic presynaptic terminals, reuptakes the transmitter from the synaptic cleft back into the neurons^15^,^25^. Thus, DAT is a crucial molecule in regulating synaptic levels of dopamine, and consequently, in determining the temporal duration of dopamine actions at local neural circuits. In addition, the D2 receptors that are expressed in dopaminergic neurons are termed autoreceptors, as these receptors potentially regulate negative feedback^26^,^27^,^28^. This regulatory process can reduce firing in dopaminergic neurons, and consequently slow the synthesis and release of dopamine. In the present study, we took advantage of the modest changes in dopamine levels caused by the genetic reduction of DAT or the pharmacological inhibition of D2 receptors *via* low dose of D2 antagonists. This enabled us to assess the functional consequences of dopamine imbalance using a set of behavioral paradigms corresponding to temporal association memory and working memory.

## Results

### Verification of baseline behaviors in DAT^+/−^ mice

To identify a separable role of dopaminergic transmission in temporal association memory, we chose a strain of hyperdopaminergic mutant mice with reduced DAT expression. The use of complete DAT knockout mice^25^ is complicated by a growth retardation phenotype and robust locomotor hyperactivity. Furthermore, the homozygote of DAT knockdown mice^15^, hereafter referred to as DAT^-/-^, have only 10% the level of wild-type (WT) DAT expression. Thus, these mice do not exhibit the growth retardation phenotype and possess normal home cage activity. However, they display hyperactivity and impaired response habituation to novel environments. As we sought to discern the dopaminergic role in temporal association memory from other potentially confounding behavioral aspects such as locomotion hyperactivity^15^, we chose to use hemizygotes of DAT knockdown mice, denoted by DAT^+/−^. In principle, these mice carry more modest changes in dopamine levels compared with the complete DAT knockouts^25^ and the homozygotes of DAT knockdown^15^ mice. They display normal locomotor activity, motor coordination, anxiety, and recognition memory compared with the WT mice^29^.

**Figure 1:**
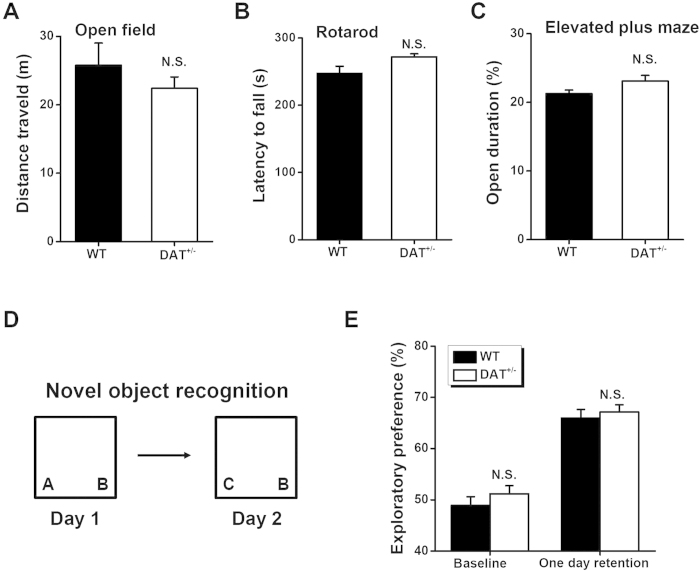
Basal behavioral performance in WT and DAT^+/−^ mice. (**A**) Total distance of the mice moved in the 10 min open field test. (**B**) Latency to fall from the rotated rod in the test one day after training. (**C**) Ratio (%) of time spent on the open arms during the elevated plus maze test. (**D**) Diagram for the novel object recognition task. (**E**) The exploratory preference of mice in the one day retention of novel objects recognition tasks. n = 10 for each group. N.S., not significant, WT *vs.* DAT^+/−^, by unpaired Student’s *t*-test.

Prior to commencing our behavioral studies, we verified baseline behaviors in DAT^+/−^ mice in terms of locomotion, emotion, and memory (Fig. 1). In the open field test, the DAT^+/−^ mice traveled a similar overall distance in the open field arena compared with that of the WT mice (Fig. 1A, *P* > 0.05), indicating unchanged basal locomotor activity in DAT^+/−^ mice. In addition, the DAT^+/−^ mice performed equally to the WT mice on the accelerating rotarod test (Fig. 1B, *P* > 0.05), confirming normal motor coordination. Moreover, the DAT^+/−^ mice spent a comparable amount of time in the open arms of the elevated plus maze compared with WT mice (Fig. 1C), indicating similar levels of anxiety in DAT^+/−^ and WT mice.

Next, to distinguish any differences in basal recognition memory between WT and DAT^+/−^ mice, we performed novel object recognition memory tests. We introduced two objects, A and B, into two distinct quadrants of the open field and recorded the amount of time that the mice spent near the objects during a 5-min period (Fig. 1D). The DAT^+/−^ mice spent a similar amount of time in the proximity of both objects compared with the WT mice, and both genotypes showed no preferences for either object A or B (*P* > 0.05, Fig. 1E). We then tested object recognition 24 hr later (Fig. 1D), by replacing one of the original objects (A) with a new object (C). Both WT and DAT^+/−^ mice showed a significantly greater preference for the new object, with no significant differences between the two strains (*P* > 0.05, Fig. 1E). This indicates that DAT^+/−^ mice have normal object recognition memory. Together, our finding indicate that, compared with WT mice, DAT^+/−^ mice exhibit normal performance in terms of baseline behaviors including locomotion, motor coordination, anxiety, and basal learning and memory.

### Impaired trace fear learning in DAT^+/−^ mice

**Figure 2:**
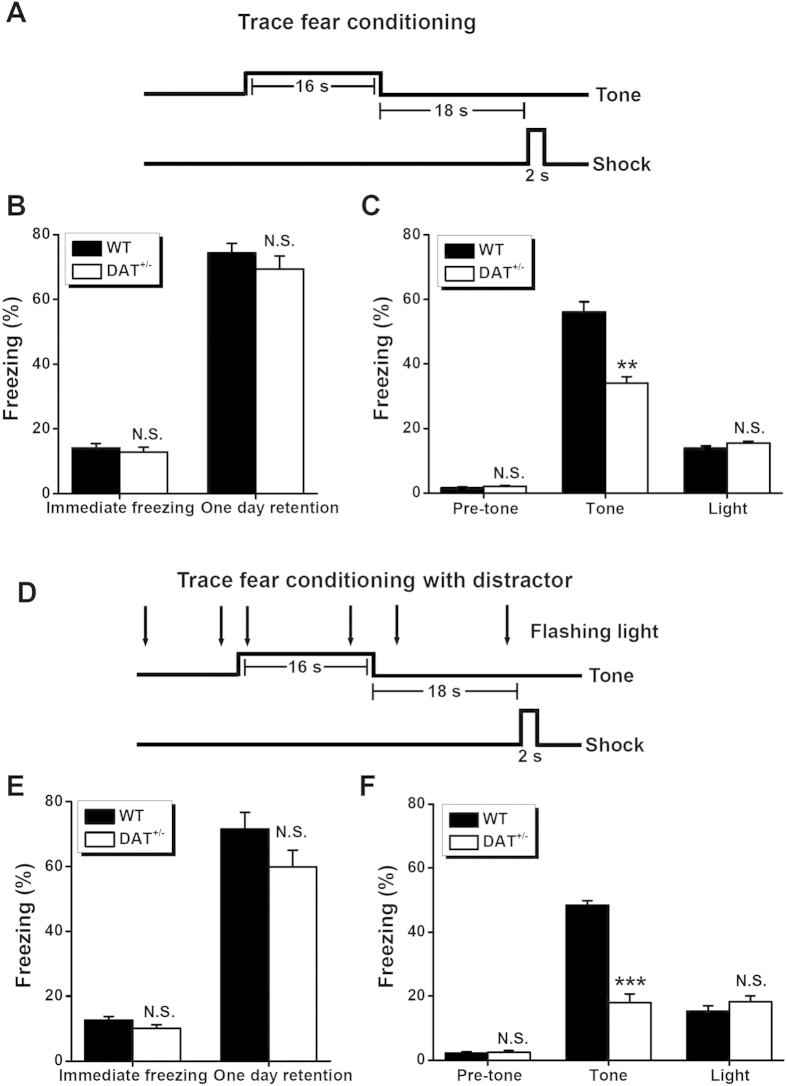
Trace fear learning in WT and DAT^+/−^ mice. (**A**) Schematic view of trace fear conditioning procedure. (**B**) Percentage of time spent freezing during the immediate freezing and one day retention tests for the contextual fear memory. (**C**) Percentage of time spent freezing in pre-tone, tone and light tests for the cued memory without a distractor. n = 9-10 for each group. N.S., not significant, **P < 0.01, WT *vs.* DAT^+/−^, by unpaired Student’s *t*-test. (**D**) Schematic view of trace fear conditioning procedure with a flashing light as the distractor. (**E**) Percentage of time spent freezing during the immediate freezing and one day retention tests for the contextual fear memory with a distractor. (**F**) Percentage of time spent freezing in pre-tone, tone and light tests for the cued memory with a distractor. n = 9-10 for each group. N.S., not significant, ***P < 0.001, WT *vs.* DAT^+/−^, by unpaired Student’s *t*-test.

To investigate whether dopamine regulation plays a role in non-spatial temporal association, we subjected the DAT^+/−^ and control mice to trace fear conditioning. Trace fear conditioning is a particular version of associative auditory fear learning, wherein a neutral conditioned stimulus (CS, i.e., a tone), when paired with an aversive unconditioned stimulus (US, i.e., a foot shock), results in a tone-driven conditioned fear response. The presence of a fear response demonstrates that an animal has formed a memory about an aversive stimulus. When a time gap (i.e., 18 s) is introduced between the end of the CS and the start of the US (Fig. 2A), the animal must rely on temporal association memory^1^,^7^,^30^. Notably, the mutant mice exhibited a similar level of freezing behavior compared with WT controls during the contextual fear test (Fig. 2B), but froze significantly less during the cued fear test (Fig. 2C) 1 day after the trace fear conditioning protocol. This implies that the DAT^+/−^ mice possessed a deficiency in trace fear learning. Importantly, the amount of immediate freezing observed after training was similar between the two strains (Fig. 2B), indicating that mutant mice have normal expression of freezing behavior. In addition, pre-tone freezing, which reflected the level of fear exhibited by the mice when they were put into a novel chamber for 2 min prior to CS tone exposure, was extremely low (and comparable) in both the DAT^+/−^ mice and WT controls (Fig. 2C). This verified that the two strains engaged in similar behaviors when subjected to a novel environment, and echoed the behavioral results from the open field test, shown above (Fig. 1A). We found both groups of animals that did not see the flashing light during training to have low freezing levels when exposed to the flashing light as a test stimulus (Fig. 2C), strengthening the specificity of the acquired fear memory. Collectively, these results suggest that a modest elevation of dopamine selectively impairs the temporal association process underlying trace fear conditioning without affecting basal emotional tone or basal memory acquisition abilities.

### Distraction aggravates impaired trace conditioning in DAT^+/−^ mice

As attention-distracting stimuli are known to interfere with the trace associative conditioning paradigm in rodents^30^ and humans^31^, we further evaluated trace conditioning in DAT^+/−^ mice in the presence of a distraction. We introduced a flashing light as a distractor during the conditioning period (Fig. 2D). We found that this distraction significantly disrupted the level of freezing in response to the cued tone in WT animals (56.1 ± 3.1% *vs.* 48.4 ± 1.4% for cued freezing in WT mice without and with the distractor, respectively, n = 9 each group, *P* < 0.05, Student’s *t*-test, Fig. 2C,F), although it did not affect the immediate freezing after training (12.6 ± 1.1% *vs.* 14.1 ± 1.4% for the immediate freezing in the WT mice after training without and with the distractor, respectively, n = 9 each group, *P* > 0.05, Student’s *t*-test, Fig. 2B,E), contextual fear freezing (74.4 ± 2.9% *vs.* 71.5 ± 5.1% for the contextual fear freezing in the WT mice 1 day after training without and with the distractor, respectively, n = 9 each group, *P* > 0.05, Student’s *t*-test, Fig. 2B,E), pre-tone freezing (1.6 ± 0.3% *vs.* 2.2 ± 0.5% for pre-tone freezing in the WT mice 1 day after training without and with the distractor, respectively, n = 9 each group, *P* > 0.05, Student’s *t*-test, Fig. 2C,F), or the freezing response to the flashing light (13.9 ± 0.7% *vs.* 15.2 ± 1.7% for flashing light-induced freezing in the WT mice 1 day after training without and with the distractor, respectively, n = 9 each group, *P* > 0.05, Student’s *t*-test, Fig. 2C,F). These observations were consistent with those of a previous report^30^, confirming that the flashing light, presented during training, acted as a distractor but not as a cue that was competing with the CS tone.

Notably, when the flashing light was presented as a distractor, it prominently aggravated the impaired trace-conditioning paradigm in the DAT^+/−^ mice. Specially, compared with the WT mice, they showed a higher degree of freezing in response to the cued tone (34.0 ± 2.0% *vs.* 18.0 ± 2.7% for the cued freezing level in the DAT^+/−^ mice without and with the distractor, respectively, n = 10 each group, *P* < 0.001, Student’s *t*-test, Fig. 2C,F). As with the WT animals, the distractor did not affect immediate freezing after training (10.1 ± 1.1% *vs.* 12.8 ± 1.5% for immediate freezing in the DAT^+/−^ mice after training without and with the distractor, respectively, n = 10 each group, *P* > 0.05, Student’s *t*-test, Fig. 2B,E), contextual fear freezing (69.5 ± 3.9% *vs.* 59.9 ± 5.2% for contextual fear freezing in the DAT^+/−^ mice 1 day after training without and with the distractor, respectively, n = 10 each group, *P* > 0.05, Student’s *t*-test, Fig. 2B,E), pre-tone freezing (2.0 ± 0.3% *vs.* 2.5 ± 0.5% for pre-tone freezing in the DAT^+/−^ mice 1 day after training without and with distractor, respectively, n = 10 each group, *P* > 0.05, Student’s *t*-test, Fig. 2C,F), or the freezing response to the flashing light (15.5 ± 0.6% *vs.* 18.2 ± 1.8% for flashing light-induced freezing in the DAT^+/−^ mice 1 day after training without and with the distractor, respectively, n = 10 each group, *P* > 0.05, Student’s *t*-test, Fig. 2C,F). Our comparison between WT and DAT^+/−^ mice with respect to freezing level after trace fear conditioning with a distractor revealed only a significant difference in the amount of the fear response to the cued tone (Fig. 2F), but no significant differences for any other stimuli (Fig. 2E,F). Together, it appears that attention-distracting stimuli can aggravate impaired processing of associations during trace fear conditioning in DAT^+/−^ mice. This finding supports the selective role of DAT-regulated dopamine in temporal association memory.

### Normal delay fear conditioning in DAT^+/−^ mice

**Figure 3:**
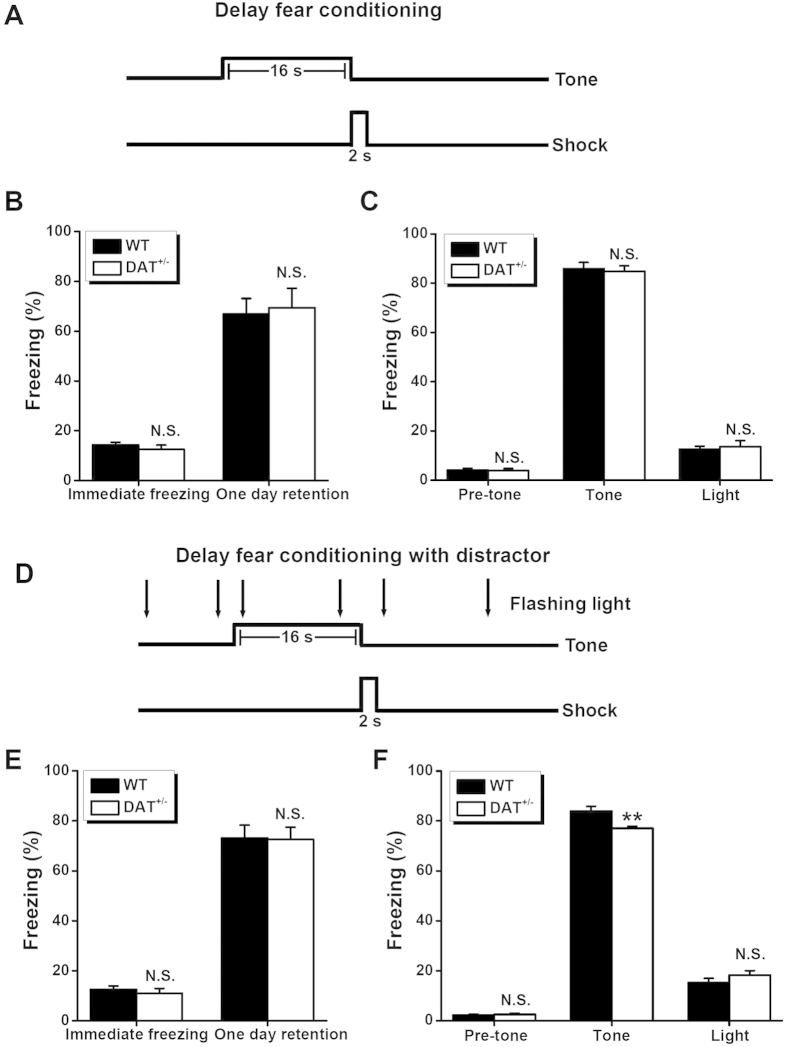
Delay fear learning in WT and DAT^+/−^ mice. (**A**) Schematic view of delay fear conditioning procedure. (**B**) Percentage of time spent freezing during the immediate freezing and one day retention tests for the contextual fear memory. (**C**) Percentage of time spent freezing in pre-tone, tone and light tests for the cued memory without a distractor. n = 9–10 for each group. N.S., not significant, WT *vs.* DAT^+/−^, by unpaired Student’s *t*-test. (**D**) Schematic view of delay fear conditioning protocol with a distractor. (**E**) Percentage of time spent freezing during the immediate freezing and one day retention tests for the contextual fear memory with a distractor. (**F**) Percentage of time spent freezing in pre-tone, tone and light tests for the cued memory with a distractor. N.S., not significant, **P < 0.01, WT *vs.* DAT^+/−^, by unpaired Student’s *t*-test.

To continue to address trace fear learning behavior in DAT^+/−^ mice compared with WT mice, we used a delay fear learning paradigm (Fig. 3A), as a control type of associative learning. This enabled us to further assess the specificity of temporal association memory^1^,^7^,^30^. In the task, a CS (i.e., auditory tone) is immediately followed by a foot shock (US). We found that the DAT^+/−^ mice behaved similarly to the WT control mice in the delay fear conditioning task. The two strains of mice showed similar contextual (Fig. 3B, *P* > 0.05) and cued (Fig. 3C, *P* > 0.05) fear memory 1 day after delay fear conditioning, in addition to immediate freezing after conditioning (Fig. 3B, *P* > 0.05), pre-tone freezing (Fig. 3C, *P* > 0.05), and freezing in response to the flashing light as an independent stimulus (Fig. 3C, *P* > 0.05). Collectively, the DAT^+/−^ mice, with their modest elevation in dopamine levels, exhibited normal performance in the delay fear conditioning task. This was in contrast to the observed impaired trace fear conditioning. This finding supports the notion of selective participation of dopamine modulation in temporal association memory.

### Disruption of delay fear conditioning by distraction in DAT^+/−^ but not WT mice

Next, we evaluated the effects of using the flashing light as a distraction, interfering with conditioned fear in the delay learning paradigm (Fig. 3D). When the flashing light was introduced as a distractor during the delay fear conditioning period (Fig. 3D), the WT mice demonstrated freezing that was comparable to that exhibited in the absence of the distractor. This was the case for all fear indices (immediate freezing: 14.4 ± 1.0% *vs.* 12.5 ± 1.3%; contextual freezing: 66.9 ± 6.2% *vs.* 73.1 ± 5.2%; pre-tone freezing: 4.1 ± 0.7% *vs.* 2.2 ± 0.5%; cued tone freezing: 85.9 ± 2.5% *vs.* 83.9 ± 2.0%; light freezing: 12.6 ± 1.2% *vs.* 15.2 ± 1.9%, without and with the distractor, respectively, n = 9 each group, all *P* > 0.05, Student’s *t*-test, Fig. 3B,C,E,F). This was consistent with the findings of a previous study^30^, indicating that delay fear conditioning requires less attention compared with trace fear learning, and therefore resists to the distraction during the learning period.

However, the DAT^+/−^ mice appeared to have been slightly but significantly distracted by the flashing light in the cued fear test (84.9 ± 2.1% vs. 77.1 ± 0.8% for the cued freezing without and with the distractor, respectively, n = 10 each group, *P* < 0.05, Student’s *t*-test, Fig. 3C,F). This was not the case for the other fear indices (immediate freezing: 12.5 ± 1.7% *vs.* 11.0 ± 2.0%; contextual freezing: 69.4 ± 7.9% *vs.* 72.5 ± 4.9%; pre-tone freezing: 3.9 ± 0.9% *vs.* 2.5 ± 0.5%; light freezing: 13.6 ± 2.5% *vs.* 18.2 ± 1.8% without and with the distractor, respectively, n = 10 each group, all *P* > 0.05, Student’s *t*-test, Fig. 3B,C,E,F). Thus, when comparing WT and DAT^+/−^ mice with respect to freezing after delay fear conditioning with a distractor, we found a unique difference in the fear in response to the cued tone (Fig. 3F), but not to other stimuli (Fig. 3E,F). These results indicate that, compared with the WT mice, DAT^+/−^ mice are more responsive to distraction. This was the case even in the delay fear learning task, which requires less attention^30^. This characteristic would undoubtedly impair the associative process during learning, regardless of whether there existed a temporally continuous or discontinuous concurrence between the CS and US.

### Inhibition of dopamine D2 receptor causes defects in trace conditioning

**Figure 4:**
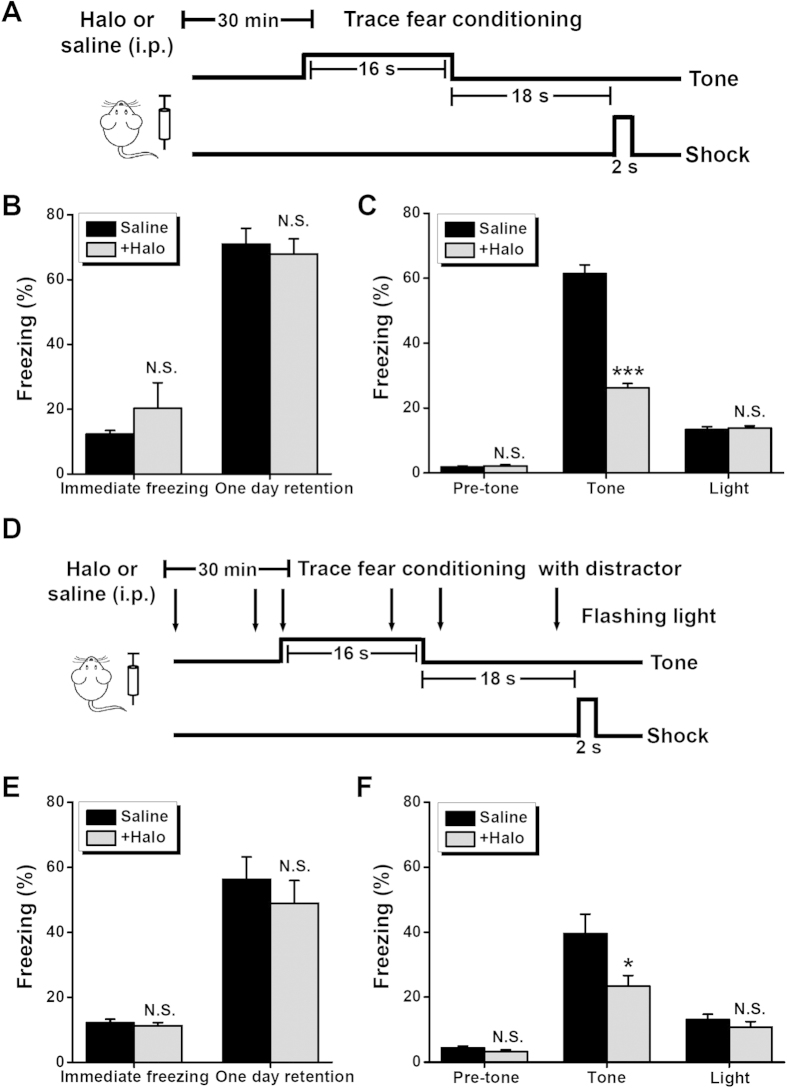
Effects of haloperidol (Halo) on trace fear learning. (**A**) Schematic view of trace fear conditioning procedure in the presence of drug application (Halo, 0.002 mg/kg of body weight in saline). (**B**) Percentage of time spent freezing during the immediate freezing and one day retention tests for the contextual fear memory. (**C**) Percentage of time spent freezing in pre-tone, tone and light tests for the cued memory without a distractor. (**D**) Schematic view of trace fear conditioning procedure with a distractor in the presence of drug application (Halo, 0.002 mg/kg of body weight in saline, i.p.). (**E**) Percentage of time spent freezing during the immediate freezing and one day retention tests for the contextual fear memory with a distractor. (**F**) Percentage of time spent freezing in pre-tone, tone and light tests for the cued memory with a distractor. In above experiments, n = 10 each group. N.S., not significant, *P < 0.05, ***P < 0.001, saline *vs.* Halo, by unpaired Student’s *t*-test.

In our exploration of the mechanisms underlying the modulation of dopamine in temporal association memory, we sought to restore or phenocopy the deficiency observed in DAT^+/−^ mice using a pharmacological approach. A low dose of the dopamine antagonist haloperidol has been found to be useful in relieving lost synaptic plasticity, cognitive inflexibility, and specific deficits in pattern completion under partial cue conditions in genetic models of hyperdopaminergia^29^,^32^. A low dose of haloperidol may be able to somewhat dampen the effect of elevated dopamine, for instance, if the phenotypes in animal models of hyperdopaminergia^29^,^32^ resulted from the excessive activation of postsynaptic D2 receptors. In such cases, the heterozygous mice may have insufficient dopamine reuptake owing to the loss of one allele from the normal DAT gene. Unexpectedly, when we gave the WT mice a low dose of haloperidol (0.002 mg/kg of body weight, i.p.) 30 min before the trace fear conditioning procedure (Fig. 4A), the amount of cued freezing after 1 day of retention was significantly lower compared with WT mice that had only received the treatment vehicle (i.e., saline). In this case, immediate freezing after fear conditioning, contextual freezing, pre-tone freezing, and freezing in response to the flashing light as an independent stimulus all remained intact (Fig. 4B,C). That a phenocopy of DAT^+/−^ mice can be generated *via* the pharmacological application of a D2 antagonist implies a shared mechanistic consequence. Considering the functional expression of D2 autoreceptors in dopaminergic neurons that exert negative feedback regulation^26^,^27^,^28^ and consequently reduce dopamine release, we favored the following hypothesis: preferential inhibition of the D2 autoreceptor over the postsynaptic D2 receptor *via* haloperidol leads to a phenotype similar to that seen in a modest animal model of hyperdopaminergia (i.e. DAT^+/−^ mice) during trace fear conditioning.

To further investigate the hypothesis that the potential inhibition of D2 autoreceptors with a low dose of haloperidol causes the observed deficits in temporal association memory, we repeated the above set of experiments with WT mice treated with haloperidol. Again, our goal was to assess the effects of a flashing light as a distractor on conditioned fear in the trace learning paradigm (Fig. 4D). When the flashing light was introduced as a distractor during the trace fear conditioning period (Fig. 4D), the application of haloperidol 30 min before conditioning further deteriorated the cued fear compared with the application of saline. However, haloperidol did not affect immediate freezing after fear conditioning, contextual freezing, pre-tone freezing, or flashing light-induced freezing (Fig. 4E,F). The results from this test battery were similar to those obtained from the DAT^+/−^ mice, i.e., aggravated impairment of trace conditioning in response to distraction (Fig. 3D-F). Therefore, it appears that the precise regulation of dopamine is critical for functional temporal association memory, regardless of the absence or presence of featured distractors. This is evidenced by the selective impairment of trace fear conditioning in mice with a dopamine imbalance caused by either insufficient dopamine reuptake (i.e., DAT^+/−^ mice) or haloperidol-induced D2 autoreceptor inhibition.

### Impaired working memory in DAT^+/−^ mice or *via* D2 receptor inhibition

**Figure 5:**
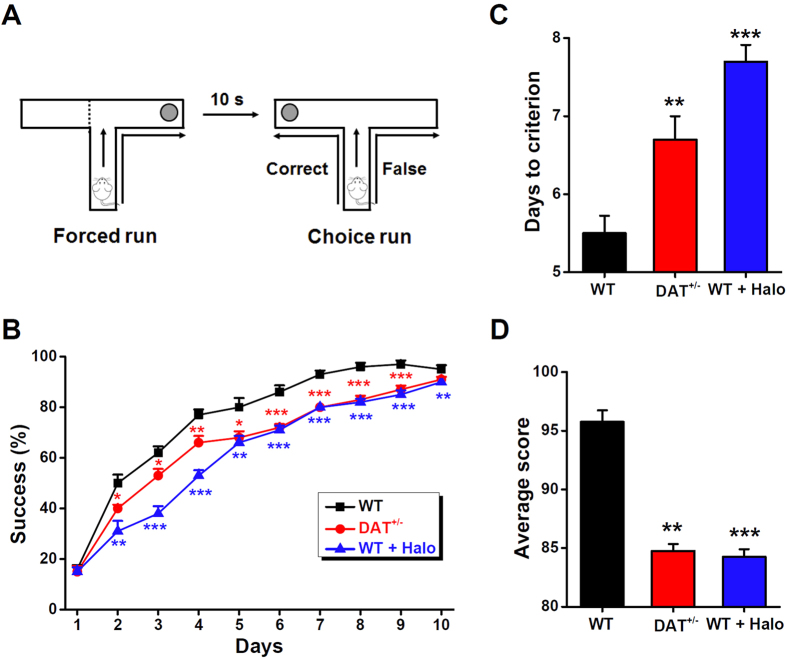
Impaired working memory in DAT^+/−^ mice or by D2 receptor inhibition. (**A**) Schematic view of a delayed non–matching-to-place version of water T-maze task. (**B**) Percentage of correct choices for mice during the learning phase in the DAT^+/−^, WT mice with and without haloperidol (Halo, 0.002 mg/kg of body weight in saline, i.p.). (**C**) Days to the criterion that mouse performed correctly seven or more out of ten trials in three consecutive days. (**D**) Percentage of correct choices for average scores. n = 10 each group. *P < 0.05, **P < 0.01, ***P < 0.001, compared with the WT group, receptively, by unpaired Student’s *t*-test.

Finally, to validate the role of the dopaminergic system in temporal association memory, we subjected the control and genetically or pharmacologically manipulated mice to a delayed non-matching-to-place version of the water escape T-maze task (Fig. 5A). This task was designed to test spatial working memory^33^,^34^, another form of temporal association memory^1^. Although all of the groups of mice showed a gradual improvement in performance from day 1 to day 10, compared with the controls, the mutant and haloperidol-treated animals exhibited a significant impairment in this task over the entire 10-day period [10 trials per day, two-way repeated-measures analysis of variance (ANOVA): group, F_(2,300)_ = 113.871, *P* < 0.001; day, F_(9,300)_ = 407.241, *P* < 0.001; interaction, F_(18,300)_ = 3.766, *P* < 0.001]. A *t* test revealed a significant difference in the success ratio between WT and DAT^+/−^ animals, as well as between the control and haloperidol-treated mice, from the second to ninth days (Fig. 5B). Moreover, compared with the WT control mice, both the DAT^+/−^ and haloperidol-treated mice took a significantly higher number of days to attain a certain criterion (in which the mouse performed correctly in at least seven out of ten trials on three consecutive days, Fig. 5C), and had lower average scores (defined as the averaged percentage of correct choices during the 4 days when the mice were trained until their correct choice percentage varied by less than 10%, Fig. 5D). These analyses confirmed that both the genetic reduction of DAT allele expression in the mutant mice and D2 receptor inhibition *via* haloperidol cause significant impairments in spatial working memory. Again, considering the similarity in the phenotypes exhibited by the mice with low dose of haloperidol-induced D2 receptor inhibition and the DAT^+/−^ mice, it is likely that preferential inhibition of the D2 autoreceptor *via* haloperidol produced a modest change in the regulation of dopamine^26^,^27^,^28^. In summary, our data support a role of dopamine regulation in temporal association memory, including spatially dependent working memory and non-spatial trace fear conditioning. Specially, we manipulated dopamine by 1) altering transmitter reuptake and 2) changing the activity of the D2 autoreceptor in dopaminergic neurons.

## Discussion

The process of associating temporally discontinuous events is critical for the formation of episodic and working memories in daily life. However, the molecular and cellular mechanisms underlying this process have remained largely unknown. In the present study, we characterized the role of dopamine in temporal association memory *via* a behavioral assessment of hemizygote hyperdopaminergic mutant mice^15^,^29^ with reduced DAT expression. We observed the DAT^+/−^ mice, which had a modest dopamine imbalance, to have normal locomotion, emotion, and novel object recognition memory (Fig. 1). However, these mice exhibited a significant deficit in trace (Fig. 2) but not delay (Fig. 3) auditory fear conditioning, in addition to impaired performance in a non-matching-to-place spatial working-memory task (Fig. 5), both of which are standard paradigms used to assess temporal association memory^1^,^7^. Interestingly, our hypothesis involving the regulation of the dopaminergic system in temporal association memory was further strengthened by the generation of a phenocopy of the selective impairment in trace fear learning and spatially working memory seen in DAT^+/−^ mice *via* a low dose of D2 receptor antagonist haloperidol (Figs 4 and 5). Taken together, our results provided a novel insight into the role of dopaminergic transmission in the processing of temporal association memory among other complex brain functions^11^,^25^,^35^,^36^,^37^,^38^,^39^,^40^,^41^.

The dopamine system is made up of dopamine-releasing neurons, referred to as dopaminergic neurons, mainly located in the midbrain, including the ventral tegmental area^20^ and the substantia nigra compacta^21^. Dopamine receptor neurons, known as dopaminoceptive neurons, express either D1- or D2-like receptors. These are widely distributed throughout the brain, for instance, in the prefrontal cortex, medial temporal lobe, and hippocampus, regions known to be actively involved in working memory^22^,^23^,^24^ and temporal association memory^1^,^7^,^9^. In addition to the dopamine receptor signaling implicated in some forms of working memory^22^,^23^,^24^ (see below), the mnemonic roles of dopaminergic regulation have also been complicated by the regulated dopamine release. DAT-mediated dopamine reuptake is one such mechanism that can efficaciously terminate dopamine signaling, and thus can determine the temporal duration of dopamine actions on local neural circuits^15^,^25^. In our evaluation of the dopaminergic mechanisms underlying temporal association memory, we found that mice with altered dopamine transmission exhibited a selective deficit in trace auditory fear conditioning, which necessitates the association temporally discontinuous elements, but not in delay auditory fear conditioning, which does not require such associations. We also found such mice to have impaired performance in the non-matching-to-place spatial working-memory task. The D2 autoreceptor in dopaminergic neurons exerts a negative feedback regulation on the synthesis and release of dopamine^26^,^27^,^28^. In the present study, we generated a phenocopy of hyperdopaminergic mutant DAT^+/−^ mice in terms of impaired temporal association memory *via* a low dose of the D2 receptor antagonist haloperidol. Although the involvement of postsynaptic D2 receptor signaling in the above phenotype was not completely excluded, we suggest that D2 autoreceptor function, in addition to DAT, may essentially contribute to temporal association memory *via* a negative feedback regulation of dopaminergic neuron signaling.

The proposed dopaminergic role in temporal association memory represents a new perspective for understanding the synaptic mechanisms of temporal association memory^1^,^9^ and working memory^10^. Several recent studies have illuminated the synaptic and circuit mechanisms underlying the process of associating temporally discontinuous elements during trace learning and working memory tasks^1^,^7^,^8^. Essentially, graded persistent neuronal activity^1^,^9^,^42^,^43^,^44^ in the entorhinal cortex has been proposed as a prerequisite element for the temporal associations in trace fear learning and working memory. To decipher the role of the hippocampal-entorhinal network in the formation of episodic and working memories, investigators created a transgenic mouse line^1^ to specifically and reversibly manipulate the synaptic output from layer III of the medial entorhinal cortex (MECIII) directly to CA1. They confirmed the specific involvement of this temporoammonic pathway in the processing of temporal association memory^1^. Additionally, clusters of excitatory neurons called island cells in layer II of the entorhinal cortex (ECII) have been reported to directly project to CA1 and activate γ-aminobutyric acid (GABA)ergic interneurons that target the distal dendrites of CA1 pyramidal neurons^7^. This can lead to the suppression of the excitatory MECIII input *via* feed-forward inhibition, thus influencing the strength and duration of temporal associations in trace learning^7^. Along the hippocampal-entorhinal network, dopamine potentially exerts its regulatory influence *via* multiple lines of mechanisms. *First*, dopamine has been found to suppress the excitatory synaptic transmission of ECII neurons, dependent on both D1- and D2-like receptors^45^,^46^. This is a potential mechanism by which the strength of sensory inputs may be suppressed in response to elevated mesocortical dopamine activity. This may happen in our DAT^+/−^ mice during the behavioral tasks. *Second*, using an *in vitro* model of the slow oscillation in the medial entorhinal cortex, dopamine had been found to strongly and reversibly suppresses activity in cortical networks, specially, persistent activity in the form of periods of sustained synchronous depolarization^47^, a neural correlate of working memory. This effect was mediated through D1- but not D2-like receptors. *Third*, dopamine facilitates GABA_A_ receptor-mediated synaptic transmission in the entorhinal cortex in an unexpected manner, i.e., this facilitation is independent of dopamine receptors, and instead relies on α1 adrenoreceptors^48^. Dopamine in the entorhinal cortex produces an inhibitory impact on network activity. In hyperdopaminergic DAT^+/−^ mice, it seems likely that elevated dopamine would disrupt the appropriate hippocampal-entorhinal network signaling that is necessary for temporal association memory. *Fourth*, in the inner part of the molecular layer of CA1 that corresponds to the temporoammonic pathway, dense D1 but not D2 receptor signaling has been identified^49^. This implies substantial dopaminergic regulation in the CA1 side of the hippocampal-entorhinal network^1^, which is required for temporal association memory. *Finally*, presynaptic D2 receptors in the dopamine fibers of the temporal hippocampus have been found to tightly modulate long-term depression expression and play a major role in the regulation of hippocampal learning and memory^28^. This finding provides a potential synaptic basis for the dopamine regulation of hippocampus-dependent temporal association memory, although this definite dopaminergic involvement has not been established before. Together, these data indicate that dopamine transmission influences the temporal association process, likely through a profound suppressive effect on the hippocampal-entorhinal network *via* multiple distinct molecular mechanisms.

In addition to the hippocampal-entorhinal network, the prefrontal cortex^50^ is also a primary site of dopaminergic modulation that may underlie temporal association memory. Signaling *via* dopamine receptors in the prefrontal cortex^22^,^23^,^24^ has been implicated in some forms of working memory. Importantly, different types of dopamine receptors in this region control distinct aspects of working memory. While persistent mnemonic-related activity in the prefrontal cortex is modulated by the D1 receptor^22^, the neural activities associated with memory-guided saccades in delayed working memory tasks selectively depend on the D2 receptor^23^. Furthermore, behavioral performance involving working memory is influenced by dopamine receptor manipulation in an extent-dependent way. Strikingly, stimulation of the D1 receptor in the prefrontal cortex produces an ‘inverted-U’ shaped dose-response^24^, whereby either too little or too much activation of this receptor impairs spatial working memory. We propose the following role of DAT in temporal association memory: this dopamine termination mechanism may mediate a temporally controlled requirement of dopamine, and activate dopamine receptors in the prefrontal cortex in a stepwise manner, representing the association process in working memory.

Dopamine is implicated in many serious cognitive disabilities and associated with a range of neurodevelopmental disorders^51^. Thus, the identified dopaminergic mechanism in temporal association memory may provide novel insights leading to new clinical developments. According to the *Diagnostic and Statistical Manual of Mental Disorders*, fifth edition^5^, ADHD is the most commonly diagnosed neurodevelopmental disorder in children. ADHD is frequently associated with learning disabilities^51^, and often extends into adulthood, with a life-long effect on cognitive and social functioning^52^,^53^. Unfortunately, comprehensive studies investigating learning and memory in people with ADHD are limited. Clinically, DAT-gene alterations have been associated with ADHD^53^,^54^. In the laboratory, both complete DAT knockout mice^25^ and the homozygotes of DAT knockdown mice^15^, exhibit behavioral impairments similar to those of ADHD patients, including locomotion hyperactivity and abridged habituation to novel environments. Using the hemizygote of DAT knockdown mice (i.e., DAT^+/−^), we previously demonstrated that modest changes in DAT function are associated with normal basal learning, consolidation, and memory recall under full cue conditions, but lead to specific deficits in pattern completion under partial cue conditions^29^. Based on the results of the present study, a selective impairment in temporal association memory can be added to the list of behavioral phenotypes exhibited by DAT mutant mice. Thus, a similar abnormality is likely to also occur in ADHD patients, which then might be considered as a potential diagnostic criteria to discern patients from normal subjects in the future.

Given the extensive evidence indicating that dopamine is essential for attention^39^,^55^ and data suggesting that the prefrontal structure is abnormal in ADHD patients^56^,^57^,^58^, it is possible that both attention and prefrontal function play a role in the temporal association of trace learning and working memory tasks through DAT-mediated dopamine regulation. Hence, the temporal association memory deficits observed in the DAT^+/−^ mice correspond to an inability to meet the increased attentional demands during the association of temporally discontinuous elements as a result of synaptic dopamine disturbance. Consistent with the notion that trace but not delay fear conditioning requires attention in mice^30^, the DAT^+/−^ mice behaved normally during delay fear learning, but exhibited a significant deficiency in trace fear learning. While in WT mice the distractor disturbed trace but not delay fear learning^30^, in the DAT^+/−^ mice, the distraction not only aggravated the impairment in trace conditioning, but also disrupted delay fear learning. This suggests that in the mutant mice, a fragile attentional process has occurred for the impairment during associative conditioning in response to disruption. Considering that the anterior cingulate cortex plays a pivotal role in attention and is required for trace over delay fear conditioning^30^, we propose a potential synaptic regulation by dopamine on the anterior cingulate cortex to underlie the attentional process. Nevertheless, we have established a significant dopaminergic role in temporal association memory. These findings may guide further studies regarding the cognitive mechanisms of episodic and working memories, in addition to illuminating the pathophysiological implications of dopamine-related disorders, thus influencing the development of mechanism-based behavioral therapy.

## Methods

### Animals

All animal procedures were carried out in accordance with the guidelines for the Care and Use of Laboratory Animals of Shanghai Jiao Tong University School of Medicine (Policy Number DLAS-MP-ANIM.01–05) and approved by the Institutional Animal Care and Use Committee [Department of Laboratory Animal Science (DLAS), Shanghai Jiao Tong University School of Medicine]. The DAT^+/−^ mice were a generous gift from the laboratory of Prof. Xiaoxi Zhuang at the University of Chicago, USA. Breeding and genotyping of DAT^+/−^ mice were the same as described^15^. All mice were maintained under standard conditions (12 hr light/dark cycle with free access to food and water). Mice were acclimatized to the testing room for at least 1 hr before all behavioral experiments. All experiments were performed in a blind manner to the genotype or the treatment of each animal.

### Fear conditioning

Fear conditioning was performed modified from a previously described procedure^30^. Both training and testing were carried out under dim light illumination conditions. On day 1, mice were brought to the training room and placed individually in the conditioning boxes for 20 min and then returned to their home cages. On day 2, mice received a 20-min baseline period in the conditioning boxes and then six trials of delay and trace fear conditioning. In delay conditioning, a foot shock (2 s at 0.5 mA) was delivered immediately after a tone (85 dB, 2 kHz, 16 s). The time between the end of the tone and next tone was 198 s. In trace conditioning, the shock was delivered 18 s after the cessation of the tone. For animals in the distraction conditions, the presentation of a distractor commenced 1 min before the first tone–shock pairing. The distractor consisted of a flashing white light (250 ms on/off for 3 s, 8 lux). The interstimulus interval sequence was randomly chosen from 5, 10, 15, or 20 s by computer and the same sequence was used for all animals. The distractor sequence was co-terminated with the final shock presentation. Thirty seconds after the last foot shock, mice were taken out of the training chambers and put back into their home cages. The freezing during this 30 s was recorded as immediate freezing. On day 3, mice were first brought to the training room and placed in the training chambers for 3 min contextual retention test. The mice were then brought to the testing room and placed in the testing chambers, receiving tone and light tests. The time between the tone and light tests was 5 min, and the order of tests was counterbalanced for each animal. In the tone test, three trials of tone testing were presented after 3 min of baseline. Each trial consisted of a tone (85 dB, 2 kHz, 30 s) followed by an interval (60 s). In the light test, the flashing light (250 ms on/off for 30 s) was used instead of the tone. The behavior of mice was videotaped throughout the session and later analyzed.

### Water T maze

A delayed alternation task^33^,^34^ in the water escape T-maze was used to evaluate working memory. Briefly, the water T maze was a grey Plexiglas T-maze pool (start arm: 47 × 10 cm; goal arms: 35 × 10 cm) filled with water (23 ± 1 °C) and white with titanium dioxide, and located in a room without visual cues that could be used by the animals to guide their response. The delayed alternation task in the water T maze consisted of a pseudorandom sequence of ten discrete trial pairs of forced-choice runs. Each trial pair consisted of a forced run in which animals were given access to only one side arm, where a submerged platform to escape from the water was located, followed by a choice run in which animals have to learn to alternate in order to find the submerged platform in the opposite arm it had entered during the previous forced run. The retention intra-trial interval (delay) between the forced run and the choice run was set at 10 s and the inter-trial interval between trial pairs was set at 30 s. A different, pseudorandom sequence (generated from https://www.random.org) of forced runs was used every day (for example, L-R-L-L-R-L-R-R-L-R), which was used for all animals tested that day. Animals were trained after they performed correctly seven or more out of ten trials (> 70% correctness) for three consecutive days. Days to reach this criterion (days of training) were used as an index of learning. Then, animals were tested until their correctness score varied less than 10% in four consecutive days. The average of the score in these 4 days was used as an index of performance. Repeated measures ANOVA and unpaired student’s *t*-test, as specified in the text, were used to compare the behavioral performance from the different genotypes or treatments.

### Open filed test

The protocols were according to previously described^59^. We conducted the open field test in a square Plexiglas apparatus (40 × 40 × 35 cm) under diffused lighting. A digital camera was set directly above the apparatus. Images were captured at a rate of 5 Hz and quantified using the Ethovision video tracking system (Noldus Information Technology, Wageningen, Netherlands). Mice were gently placed in the square and allowed to explore freely for 10 min. After each trial, the apparatus was cleaned and the animal returned to the home cage.

### Rota-rod test

For the measurement of the Rota-rod test, the mice were placed on an accelerating rotating wooden-rod (Ugo Basile, Como, Italy). The rod was 12 inch long and 1 inch in diameter. The initial rotation speed was at 4 rpm and then steadily accelerated to 40 rpm. The performance was measured by the amount of time (in seconds) that mice managed to remain on the rotating rod one day after training.

### Elevated plus maze test

The protocols were followed as described^60^. The elevated plus maze was made of black plastic material, and consisted of four arms (two open without walls and two enclosed by 15.25 cm high walls) 30 cm long and 5 cm wide. Each arm of the maze was attached to sturdy metal legs such that it was elevated 40 cm above the floor. Activity was recorded by a digital camera suspended from the ceiling. Testing took place under dim light during the light phase (between 07:00 a.m. and 07:00 p.m.). The maze was cleaned with 10% alcohol between tests. On the test day, animals were brought into the testing room in their home cages. Animals were placed individually in the center of the maze facing the enclosed arms, and behavior was recorded for 5 min. The time spent on the open arms and closed arms were recorded and analyzed.

### Novel-object recognition task

The experimental protocol was the same as described previously^59^. Mice were individually habituated to an open field box (40 × 40 × 35 cm) before test. In the first day, two different objects (A and B) were used as acute novelty and placed into two distinct quadrants of the open field and each mouse was allowed to explore for 5 min. The object recognition was tested 24 hr later. The mice were placed back into the same box, in which one of the familiar objects (A) was replaced by a novel object (C), and allowed to explore freely for 5 min. The time spent in the proximity of the object (the quadrant with object) was measured using the Ethovision video tracking system (Noldus Information Technology, Wageningen, Netherlands).

### Statistical analyses

Data were analyzed with Student’s *t* test or one-way or two-way repeated-measures ANOVA, followed by Fisher’s LSD post hoc comparisons, where appropriate. ^*^P < 0.05, ^**^P < 0. 01, and ^***^P < 0.001 represent significant differences. All values in the text and Figures represent means ± SEM. Data analyses were performed using SPSS statistical program version 10.0.

## Additional Information

**How to cite this article**: Deng, S. *et al.* A behavioral defect of temporal association memory in mice that partly lack dopamine reuptake transporter. *Sci. Rep.* **5**, 17461; doi: 10.1038/srep17461 (2015).
